# Plasma microRNAs reveal a schizophrenia patient subgroup with high inflammation and severe symptoms

**DOI:** 10.1192/j.eurpsy.2025.821

**Published:** 2025-08-26

**Authors:** T. Miyano, T. Mikkaichi, K. Nakamura, Y. Yoshigae, K. Abernathy, Y. Ogura, N. Kiyosawa

**Affiliations:** 1 Translational Science Department II; 2 Translational Research Laboratories, Daiichi Sankyo Co., Ltd., Tokyo, Japan; 3 Clinical Research Department, Sirtsei Pharmaceuticals, Inc., North Carolina, United States

## Abstract

**Introduction:**

Schizophrenia is a complex and highly heterogeneous psychiatric disorder, and it is crucial to understand the different pathophysiology among patients to realize precision psychiatry.

**Objectives:**

This study aims to evaluate the potential of plasma microRNAs (miRNAs) as clinical biomarkers to stratify schizophrenia patients based on molecular profiles and understand the heterogeneous pathophysiology.

**Methods:**

We measured the Positive and Negative Syndrome Scale (PANSS) scores, which are severity scores of clinical symptoms of schizophrenia, along with levels of 179 plasma miRNAs in 26 schizophrenia patients experiencing acute psychosis. We applied hierarchical clustering analysis for the plasma samples on miRNA levels to explore patient subgroups. We then conducted miRNA set enrichment analysis, literature-based text mining and manual literature survey on characteristic miRNAs for each patient subgroup to interpret the heterogenous pathophysiology. This study has been approved by the Ethical Research Practice Committee of Daiichi Sankyo Co., Ltd.

**Results:**

The schizophrenia patients were stratified into three subgroups based on the plasma miRNA profiles. One of these patient subgroups showed a tendency to have relatively high PANSS scores. This patient subgroup was characterized by distinctively low levels of four miRNAs. The enrichment analysis revealed an enrichment of ‘Immune Response’ pathways associated with these four miRNAs. Consistent with the enrichment results, literature-based text mining confirmed that these four miRNAs were frequently associated with ‘inflammation’ and IL-1β, IL-6, and TNFα in the literature. We also identified literature-based experimental evidence demonstrating that these four miRNAs reduce IL-1β, IL-6 and TNFα. These results suggest that the patient subgroup with high PANSS scores has relatively high inflammation.

**Conclusions:**

miRNAs may potentially be clinical biomarkers that reflect both the symptoms and molecular pathology of schizophrenia, and they may be able to identify patient subgroups with relatively high inflammation. Such patient stratification based on molecular profiles is expected to be a key tool to realize precision psychiatry, e.g., prescribing right drugs for right patients.

**Disclosure of Interest:**

T. Miyano Employee of: Daiichi Sankyo Co. Ltd., T. Mikkaichi Employee of: Daiichi Sankyo Co. Ltd., K. Nakamura Employee of: Daiichi Sankyo Co., Ltd., Y. Yoshigae Employee of: Daiichi Sankyo Co., Ltd., K. Abernathy Employee of: Sirtsei Pharmaceuticals, Inc., Y. Ogura Employee of: Daiichi Sankyo Co., Ltd., N. Kiyosawa Employee of: Daiichi Sankyo Co., Ltd.

